# Comparison of Neck Circumference at the Hyoid and Thyroid Levels, Neck Circumference to Thyromental Distance Ratio, and Hyomental Distance Ratio in Predicting Difficult Laryngeal Visualization in Obese and Non-Obese Populations

**DOI:** 10.7759/cureus.78879

**Published:** 2025-02-11

**Authors:** Barathan Thirunavukkarasu, Sofia Jaswal, Harsimran S Walia, Y. K Batra

**Affiliations:** 1 Anesthesiology, Max Super Specialty Hospital, Mohali, IND; 2 Anesthesia and Critical Care, Homi Bhabha Cancer Hospital and Research Center, New Chandigarh, IND; 3 Anesthesia Critical Care and Pain, Homi Bhabha Cancer Hospital and Research Center, New Chandigarh, IND; 4 Anesthesia and Pain Management, Max Super Specialty Hospital, Mohali, IND

**Keywords:** difficult, larynx, non-obese, obese, visualization

## Abstract

Introduction: Several preoperative predictive factors for difficult visualization of the larynx (DVL) have been identified, and these factors have significant predictive value in the obese population. This prospective observational study was conducted to compare the predictive abilities of different airway predictors between obese and non-obese populations.

Material and methods: Our study included a total of 90 patients, divided into two groups based on body mass index (BMI): group O (obese) with BMI >30 kg/m² and group NO (non-obese) with BMI <30 kg/m². The patients were evaluated using the modified Mallampati grade (MMG), neck circumference at the level of the hyoid (NCh) and thyroid (NCt), thyromental distance (TMD), and hyomental distance (HMD) in both neutral and extended head positions in both groups. The modified Cormack and Lehane (MCL) grading for visualization of the larynx was observed during direct laryngoscopy prior to intubation. MCL grades 2b, 3, and 4 were considered indicative of DVL in this study.

Results: DVL was observed in 41.1% of the population, 46.7% of the obese (O) group, and 35.6% of the non-obese (NO) group. MMG was statistically significant only in the O group for predicting the DVL with a specificity of 85.1%. In the NO group, MMG ≥3 was not significant statistically and showed a specificity of 70.8%. NCh and NCt had no statistically significant difference in differentiating the DVL between obese and non-obese groups. NCt/TMD in the O group with a cut-off value of 5.135 had a sensitivity of 61.9% and a specificity of 58.3% and predicted DVL only in the obese group. Hyomental distance ratio (HMDR) was a statistically significant index in both groups and has a good predictive utility for the DVL. An HMDR ≤1.23 had a sensitivity of 68.8% and a specificity of 72.4% in the non-obese population. An HMDR ≤1.20 in the obese group had a sensitivity of 85.7% and a specificity of 91.7%.

Conclusion: The higher incidence of DVL (MCL grades 2b, 3, and 4) in the obese population when compared to the non-obese population was not statistically significant. NCt and NCh had minimal value as predictors of DVL. This study concludes that HMDR was a reliable airway assessment tool irrespective of BMI, while MMG and NCt/TMD were reliable difficult airway predictors but only in the obese population.

## Introduction

Anesthesiologists often experience unforeseen difficulty acquiring ideal exposure of the larynx while doing laryngoscopy, making endotracheal intubation difficult. Unanticipated difficult direct laryngoscopy was synonymous with difficult intubation in most patients. Complications related to difficult airway management may lead to side effects such as desaturation, aspiration, injury to the larynx and esophagus, hypoxic brain injury, cardiac arrest, and subglottic airway access insertion [1]. Recognition of pre-operative airway predictors of difficult airway is important to establish safer airway management techniques. Failure to predict and plan appropriately is the most critical factor that could lead to the catastrophic “cannot intubate cannot oxygenate” scenario [2]. While difficult endotracheal intubation is a complex interaction between the patient, clinical setting, and practitioner, the direct reason is a difficult laryngoscopy [3]. The cause of a difficult direct visualization of the larynx (DVL) is multifactorial and can be both anatomical and physiological. Obesity is a clinical condition that comprises both anatomical and physiological factors, substantially increasing the difficulty of airway management [4]. According to WHO, obesity is defined as a body mass index (BMI) >30 kg/m^2^. Fat deposition leads to enlarged neck, breasts, pharyngeal, and palatal soft tissues causing abnormal morphology of the head, neck, and torso. Several airway indices as predictors of DVL are included in routine pre-anesthetic check-ups (PAC). The search for a non-invasive and more accurate airway assessment test has given us various ultrasound-derived measurements that have been devised to predict difficult laryngoscopy [5,6]. The soft tissues of the neck, anterior to the hyoid bone and thyrohyoid membrane, need to be displaced by the laryngoscope blade during laryngoscopy. While routine pre-operative ultrasonographic airway assessment is not yet feasible everywhere, measuring the overall neck circumference at the level of the hyoid (NCh) can serve as a simpler surrogate marker. Although neck circumferences at the levels of the thyroid and cricoid cartilages have been studied, more evidence is needed to establish the significance of NCh. The modified Mallampati grade (MMG), NCt, and the neck circumference to thyromental distance (TMD) ratio are good predictors of a DVL in the obese population. However, data on their significance in the non-obese population is limited and warrants further studies. The objective of this study is to compare the predictive ability of several airway indices in obese and non-obese populations for DVL. MMG, NCt, NCt/TMD, NCh, and HMDR were measured in all patients to predict DVL.

## Materials and methods

Study design

The study was a hospital-based, prospective, observational, comparative study conducted at Max Super Speciality Hospital, Mohali, from December 2020 to December 2021. It was approved by the institutional ethics committee (reference number: TS/MSSH/MOHALI/HBPL/IEC/ANAES/20-15), and written informed consent was obtained from all patients. The patients' weight (wt) in kilograms and height (h) in meters were measured while they were standing, and BMI was calculated using the formula: wt/h². The inclusion criteria were patients aged over 18 years of either sex, undergoing elective surgery under general anesthesia, and able to provide written informed consent upon admission. Patients with a history of reactive airway disease, non-fasted state, requiring rapid sequence intubation, raised intracranial pressure, known hypersensitivity to intravenous anesthetic agents, or upper airway pathologies (such as tumors, craniofacial fractures, cervical pathology, tracheal stoma, and pregnancy) were excluded from the study. The primary objective was to compare the incidence of DVL in obese and non-obese patients undergoing general anesthesia requiring endotracheal intubation. The secondary objective was to evaluate and compare the predictive accuracy of different airway assessment indices for DVL between obese and non-obese patients.

The sample size was calculated based on previous studies that reported an incidence of DVL ranging from 6.3% to 31% in lean [7] and obese [8] patients, respectively. According to the calculations, to demonstrate the difference with 80% power (alpha = 0.05 and beta = 0.2), each group required 38 patients. However, a total of 90 patients were assigned to two groups based on the calculated BMI: group O (obese, BMI > 30 kg/m²) and group NO (non-obese, BMI <30 kg/m²).

Anesthesia technique

The day before surgery, all patients underwent a detailed history, physical examination, and group allocation. Demographic details, including age, gender, BMI (kg/m²), co-morbidities, American Society of Anesthesiologists (ASA) status, and any history of previous surgery with a difficult airway, were documented. A routine airway examination was performed. The TMD was measured with the neck fully extended and the mouth closed [9]. Using a measuring tape, the distance between the thyroid cartilage and symphysis menti was recorded. The extended hyomental distance (HMDe) was measured in the same position, between the hyoid bone and symphysis menti [10]. The Neutral Hyomental Distance (HMDn) was measured with the head and neck in a neutral position [10]. Neck circumference at the NCt and NCh were also measured [9]. MMG was noted with the patient in a sitting position, head forward, and mouth wide open with the tongue sticking out without phonation [11]. The observer positioned their eye level at the patient’s mouth. MMG was noted based on the visibility of the faucial pillars, uvula, soft palate, and hard palate. Nil per oral orders were given, with eight hours of fasting for solids and two hours for clear fluids before surgery. All patients were pre-medicated with oral pantoprazole 40 mg and alprazolam 0.25 mg on the night before surgery. Patients were informed about the study, and their approval for participation was recorded in written consent forms. On the day of surgery, the patient was shifted to the operating room, and standard ASA monitors were attached. All patients were pre-oxygenated with 100% oxygen before induction. Anesthesia was induced with an injection of fentanyl (2 mcg/kg) and propofol (2-2.5 mg/kg) for loss of consciousness, and muscle relaxation was achieved with an injection of atracurium (0.5 mg/kg).

Evaluation of outcome

After induction, bag and mask ventilation of the patient was performed using 100% oxygen for three minutes by the anesthesiologist. Direct laryngoscopy was then carried out using a Macintosh laryngoscope. The anesthesiologist performing the laryngoscopy recorded the glottic visualization according to the modified Cormack and Lehane (MCL) grading system. MCL grades 1 and 2a were considered indicative of easy visualization of the larynx (EVL), while MCL grades 2b, 3, and 4 were considered indicative of DVL. Anesthesia was maintained with sevoflurane (at a minimum alveolar concentration of 1 - 1.2) in a mixture of oxygen and medical air (1:1), supplemented with atracurium and fentanyl top-ups.

Statistical analysis

The statistical software IBM SPSS Statistics 20.0 (IBM Corporation, Armonk, NY, USA) was used for data analysis. Descriptive and inferential statistical analyses were performed in the present study. All quantitative variables were analyzed using measures of central location (mean and median) and measures of dispersion (SD). Results for continuous measurements are presented as Mean ± SD, while results for categorical measurements are presented as numbers (%). The level of significance was set at p = 0.05, with values less than or equal to 0.05 considered statistically significant. Chi-square analysis was used to assess the significance of study parameters on a categorical scale. Student's t-test (two-tailed, unpaired) was used to assess the significance of study parameters on a continuous scale between two groups. The receiver operating characteristic (ROC) curve was used to predict sensitivity and specificity with a 95% CI and a cut-off value.

## Results

Demographic data

The mean age in the O group was 46.9 ± 14.1 years, and in the NO group, it was 43.8 ± 12 years. There was no significant difference between the two groups in terms of age (p > 0.05) (Table 1). The mean height in the O group was 1.64 ± 0.08 meters, while the mean height in the NO group was 1.65 ± 0.08 meters. There was no significant difference between the two groups in terms of height (p > 0.05) (Table 1).

**Table 1:** Demographic data

Data	Obese (n = 45)		Non-obese (n = 45)		T-value	P-value
	Mean	SD	Mean	SD		
Age	46.93	14.12	43.82	12.052	1.124	0.264
Height	1.6471	0.08109	1.6533	0.8144	0.363	0.717

In the O group, 24 (53.3%) were males and 21 (46.7%) were females, while in the NO group, 19 (42.2%) were males and 26 (57.8%) were females. There was no significant difference between the two groups in terms of gender distribution (p > 0.05) (Table 2).

**Table 2:** Distribution of gender

			Gender		Total
			Male	Female	
Group	O	Count	24	21	45
		% within group	53.3%	46.7%	100.0%
	NO	Count	19	26	45
		% within group	42.2%	57.8%	100.0%
Total		Count	43	47	90
		% within group	47.8%	52.2%	100.0%
Chi-square value: 1.113; p-value: 0.291					

Distribution of modified Cormack and Lehane grade

During laryngoscopy, MCL grade 2A was observed in 15 (33%) and 22 (49%) subjects in the O and NO groups, respectively. Among the grades indicating a DVL, grade 2B was observed in 11 (24.4%) and eight (17.8%) subjects in the O and NO groups, respectively. A total of 21 (46.7%) and 16 (35.6%) subjects in the O and NO groups, respectively, had a difficult laryngoscopic view (Table 3). There was no statistically significant difference observed between the two groups, as assessed using the chi-square test (p > 0.05).

**Table 3:** Distribution of MCL grade MCL, modified Cormack and Lehane

			MCL grade					Total
			1	2a	2b	3	4	
Group	O	Count	9	15	11	7	3	45
		% within group	20.0%	33.3%	24.4%	15.6%	6.7%	100.0%
	NO	Count	7	22	8	4	4	45
		% within group	15.6%	48.9%	17.8%	8.9%	8.9%	100.0%
Total		Count	16	37	19	11	7	90
		% within group	17.8%	41.1%	21.1%	12.2%	7.8%	100.0%
Chi-square value: 3.009; p-value: 0.556								

Comparison of different indices

The mean height of the patients in the obese group who had EVL and DVL was 1.64 ± 0.7 m and 1.65 ± 0.8 m, respectively. In the non-obese group, the mean height of those who had EVL and DVL was 1.66 ± 0.8 m and 1.63 ± 0.7 m, respectively. The results obtained using an unpaired t-test showed no statistical difference between the mean height and DVL in both the obese (O) and non-obese (NO) groups (p > 0.05) (Table 4). In the NO group, the mean weight of patients with EVL and DVL was 70.7 ± 10.8 kg and 65 ± 8.5 kg, respectively. The unpaired t-test showed no significant difference (p > 0.05). However, in the O group, the mean weight of patients with EVL and DVL was 88.3 ± 11.3 kg and 97.9 ± 18.4 kg, respectively. The unpaired t-test showed a statistically significant difference (p < 0.05) (Table 4). The mean BMI of non-obese patients with EVL and DVL was 25.5 ± 2.7 kg/m² and 24.2 ± 2.8 kg/m², respectively. The unpaired t-test showed no statistically significant difference in the NO group. In the O group, the mean BMI of patients with EVL and DVL was 32.7 ± 2.1 kg/m² and 35.6 ± 4.8 kg/m², respectively. The difference was statistically significant (p < 0.05) (Table 4). The mean NCt in the O group was 38.7 ± 4.9 cm in patients with EVL and 42 ± 6 cm in patients with DVL. The difference was not statistically significant (p = 0.052). In the NO group, patients with EVL and DVL had mean NCt values of 35.7 ± 2.3 cm and 36.4 ± 2.9 cm, respectively. The unpaired t-test yielded a p-value > 0.05, indicating no statistically significant difference in the NO group (Table 4). The mean NCh in the O group for patients with EVL and DVL was 40.1 ± 4.9 cm and 43.32 ± 5.8 cm, respectively. There was no statistically significant difference in the O group (p > 0.05) using the unpaired t-test. In the NO group, the mean NCh for patients with EVL and DVL was 36.9 ± 2.3 cm and 37.6 ± 3.0 cm, respectively. With a p-value > 0.05, there was no statistically significant difference in the NO group (Table 4). The NO group had a mean NCt/TMD ratio of 4.8 ± 0.8 and 5.2 ± 1.3 for patients with EVL and DVL, respectively. No significant difference was observed in the NO group using the unpaired t-test. In the O group, the mean NCt/TMD ratio was 4.9 ± 0.4 and 5.3 ± 0.7 for patients with EVL and DVL, respectively. The unpaired t-test showed a statistically significant difference (p < 0.05) (Table 4).

**Table 4:** Comparison of different indices *Statistically significant **Highly significant BMI, body mass index; NCh, neck circumference at the level of the hyoid; NCt, neck circumference at the level of the thyroid; TMD, thyromental distance; HMDR, hyomental distance ratio; EVL, easy visualization of the larynx; DVL, difficult visualization of the larynx

Parameter	Obese						Non-obese					
	EVL (n = 24)		DVL (n = 21)		T-value	P-value	EVL (n = 29)		DVL (n = 16)		T-value	P-value
	Mean	SD	Mean	SD			Mean	SD	Mean	SD		
Height	1.6413	0.07753	1.6538	0.08640	0.514	0.610	1.6614	0.08551	1.6388	0.07384	0.890	0.378
Weight	88.34	11.393	97.90	18.479	2.111	0.041*	70.72	10.856	65.0	8.571	1.817	0.076
BMI	32.702	2.134	35.636	4.889	2.666	0.011*	25.542	2.788	24.209	2.847	1.523	0.135
NCt	38.754	4.9105	42.014	6.0464	1.995	0.052	35.717	2.3704	36.4	2.926	0.85	0.4
NCh	40.146	4.9676	43.329	5.8239	1.979	0.054	36.959	2.3944	37.681	3.0266	0.882	0.383
NCt/TMD	4.966	0.486	5.394	0.765	2.269	0.028*	4.898	0.850	5.253	1.339	1.087	0.283
HMDR	1.331	0.126	1.06	0.162	6.242	<0.001**	1.317	0.130	1.119	0.275	3.283	0.002*

**Figure 1:**
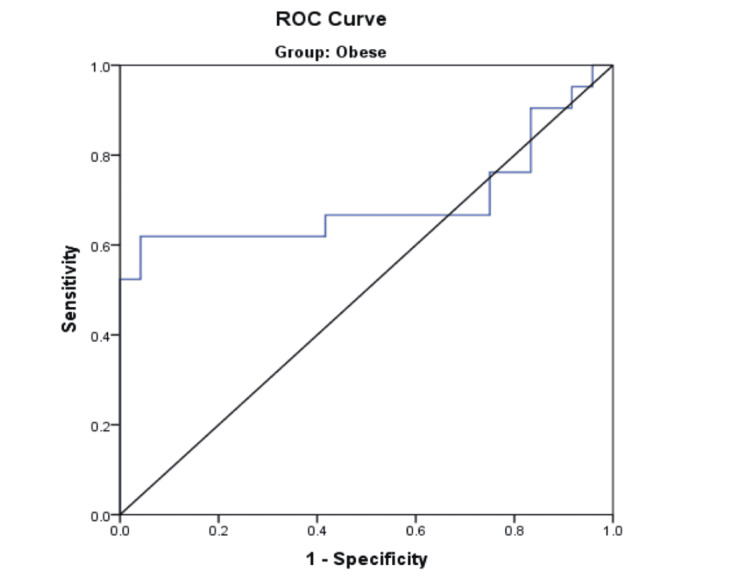
ROC curve - NCt/TMD (obese group) NCt, neck circumference at the level of the thyroid; TMD, thyromental distance; ROC, receiver operating characteristic

A ROC curve was constructed using the NCt/TMD ratio to assess its ability to predict DVL in the O group (Figure 1).

With a 95% CI, the ROC analysis yielded an area under the curve (AUC) of 0.696 at a cutoff value of 5.135. It demonstrated a sensitivity of 61.9% and a specificity of 58.3% (Table 5).

**Table 5:** AUC - NCt/TMD (obese group) and HMDR (obese and non-obese groups) *Statistically significant **Highly significant AUC, area under the curve; NCt, neck circumference at the level of the thyroid; TMD, thyromental distance; HMDR, hyomental distance ratio

	AUC							
Variable	Area	Standard error	p-value	95% CI		Cut-off	Sensitivity	Specificity
				Lower limit	Upper limit			
NCt/TMD: obese group	0.696	0.089	0.024*	0.522	0.871	5.135	0.619	0.583
HMDR: obese group	0.911	0.052	0.0001**	0.808	1.000	1.200	0.857	0.917
HMDR (non-obese group)	0.772	0.084	0.003**	0.607	0.936	1.235	0.688	0.724

**Figure 2:**
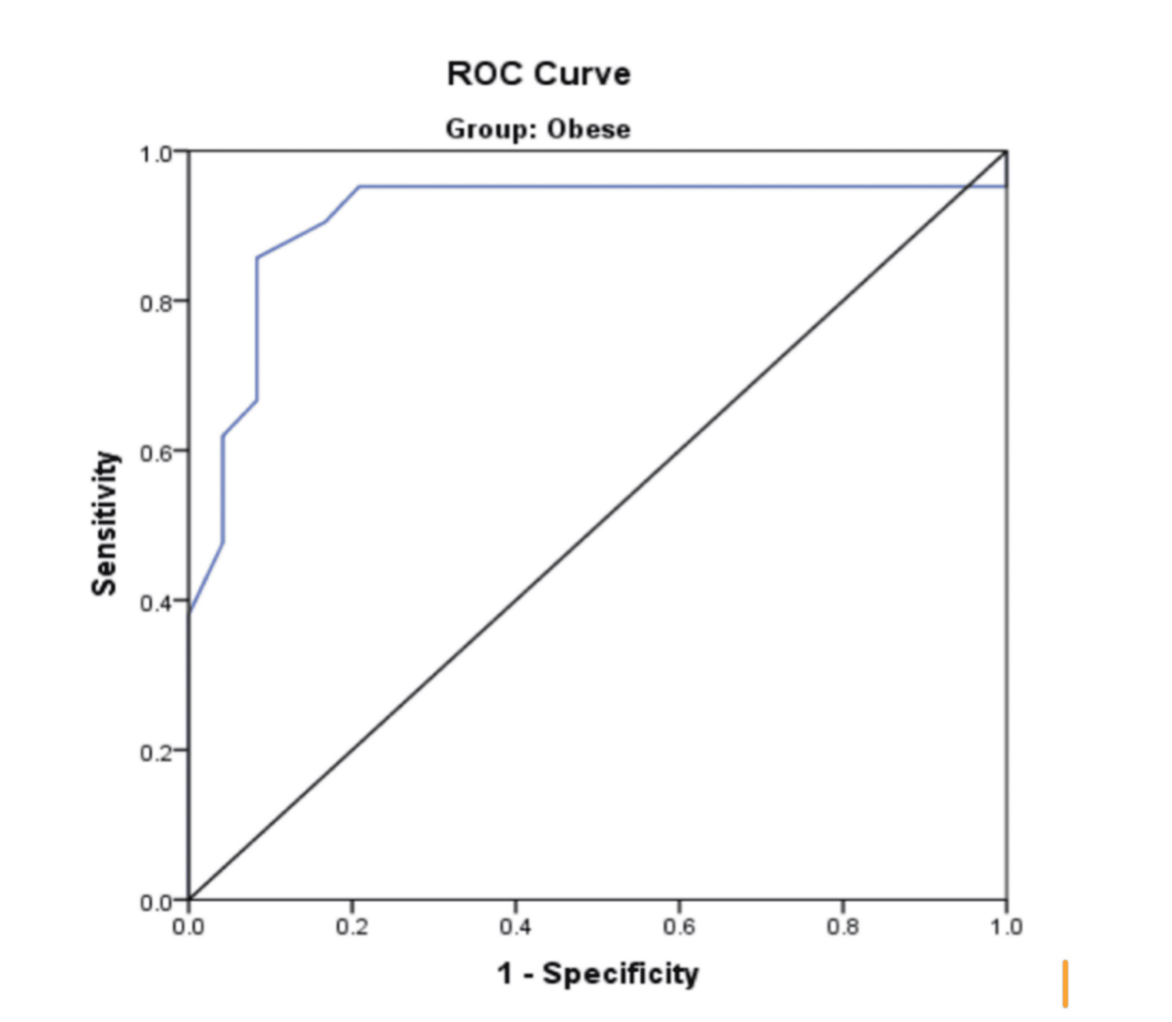
ROC curve - HMDR (obese group) HMDR, hyomental distance ratio; ROC, receiver operating characteristic

The mean HMDR of patients with EVL and DVL in the NO group was 1.31±0.13 and 1.11±0.27, respectively. This difference was statistically significant based on the unpaired t-test (p < 0.05) (Table 4). The mean HMDR in the O group for patients with EVL and DVL was 1.33±0.12 and 1.06±0.16, respectively. This difference was highly significant when applying the unpaired t-test (p < 0.001) (Table 4). In the O group, a 95% CI was applied to the ROC curve of HMDR to assess its ability to predict DVL (Figure 2).

**Figure 3:**
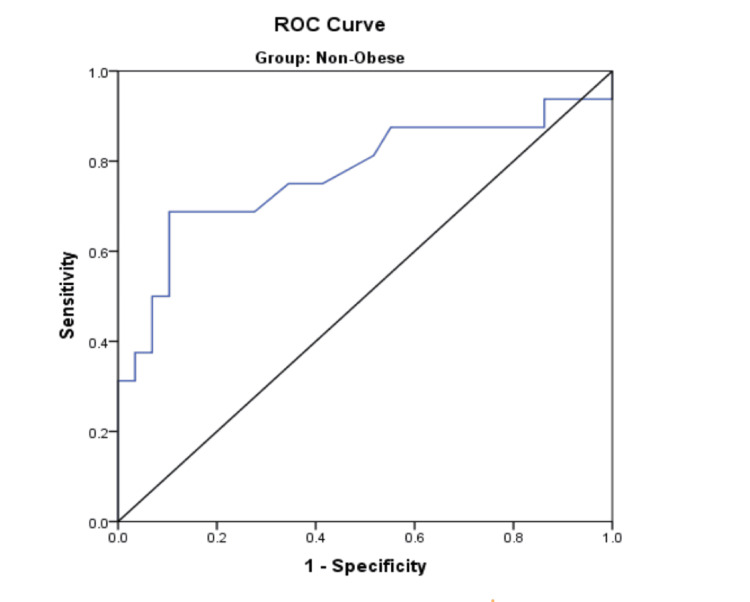
ROC curve - HMDR (non-obese group) HMDR, hyomental distance ratio; ROC, receiver operating characteristic

The AUC derived was 0.911 with a cut-off value of 1.200, and it had a sensitivity of 85.7% and specificity of 91.7% (Table 5). ROC curve analysis of the non-obese group (Figure 3) showed that HMDR had a sensitivity of 68.8% and a specificity of 72.4% with a cut-off value of 1.235. The AUC was 0.772 (95% CI: 0.607-0.904) (Table 5).

MMG grades 1 and 2 were considered predictors for EVL, while grades 3 and 4 were considered predictors for DVL. From Table 6, there were only two patients with MMG grade 4, and both belonged to the O group. In the O group, 55.5% of patients had MMG grades 1 and 2, while 44.5% had MMG grades 3 and 4. In the NO group, 75.5% of patients had MMG grades 1 and 2, while 24.5% had MMG grade 3 (Table 6). In the NO group, MMG detected DVL in only seven out of 18 patients (39%), while predicting EVL in 23 out of 27 patients (85%). Upon applying the chi-square test, there was no significant correlation between MMG and VL according to MCL grade (p > 0.05) (Table 6).

**Table 6:** MMG and MCL grade in the obese and non-obese group EVL, easy visualization of the larynx; DVL, difficult visualization of the larynx; MMG, modified Mallampati grade; MCL, modified Cormack and Lehane

		MMG		Total	Chi-square value	P-value
		MMG 3, 4	MMG 1, 2			
MCL grade (non-obese group)	DVL	7	11	18	3.389	0.05
	% within group	39%	61%	100%		
	EVL	4	23	27		
	% within group	15%	85%	100%		
Total		11	34	45		
MCL grade (obese group)	DVL	13	8	21	4.861	<0.05*
	% within group	62%	38%	100%		
	EVL	7	17	24		
	% within group	29%	71%	100%		
Total		29	25	45		

In the O group, MMG indicated difficulty in 13 out of 21 patients (62%) who had DVL, while MMG predicted an EVL (MCL 1, 2a) in 17 out of 24 patients (71%) who had EVL during direct laryngoscopy. With a p-value < 0.05 from the chi-square test, MMG showed a statistically significant correlation with VL in the O group (Table 6). Although MMG did not show a statistically significant correlation in the NO group, it had a sensitivity of 61.9% and a specificity of 70.8%. In the O group, MMG had a sensitivity of 38.8% and a specificity of 85.1%. MMG showed an overall accuracy of 66.7% in both groups (Table 7).

**Table 7:** Comparison of MMG in both groups TP, true positive; TN, true negative; FP, false positive; FN, false negative; SENS, sensitivity; SPEC, specificity; PPV, positive predictive value; NPV, negative predictive value

Test	TP	TN	FP	FN	SENS	SPEC	PPV	NPV	Accuracy
O group	7	23	4	11	38.8%	85.1%	63.6%	67.6%	66.7%
NO group	13	17	7	8	61.9%	70.8%	65%	68.1%	66.7%

## Discussion

It is important to predict and identify potential risk factors for a difficult airway and to evaluate the need for an appropriate alternative airway management strategy to minimize the risk of complications. Evaluation begins with a preliminary pre-anesthesia check-up, where an assessment of airway indices can alert the anesthesiologist to anticipate a potentially difficult airway intraoperatively. This provides time for preparation and better management. An increase in tongue size, a short neck, excessive laryngeal soft tissue, obesity, associated obstructive sleep apnea, and the position of the hyoid bone relative to the epiglottic vallecula are factors that influence direct laryngoscopy and laryngeal exposure. Conflicting literature exists on predictive factors for a difficult airway in the obese population, although obesity itself is a major risk factor. We conducted this study to compare the relationship between five pre-operative airway assessment indices and DVL in obese and non-obese populations. Our populations in both groups were comparable in age, gender, and height, with no statistically significant difference.

Modified Cormack and Lehane grade

The Yentis et al. modification of the original Cormack and Lehane grading introduced grade 2a (partial view of the glottis) and grade 2b (view of only the arytenoids with or without partial visualization of the posterior vocal cords). Their study demonstrated that among patients with DVL (grades 2b, 3, and 4), about 67% had a grade 2b view [12]. In our study, using the MCL grading, the overall incidence of DVL was observed in 37 patients (41.1%). In the O group, 21 patients (46.7%) had MCL grades 2b, 3, and 4. In the NO group, 16 patients (35.6%) had MCL grades 2b, 3, and 4. There was a higher overall frequency of DVL in our population compared to that observed by other authors. The incidence of DVL varied from 7.2% to 31%, as observed by Ajinkya et al. in 9% of 300 subjects [13], Huh et al. observed DVL in 12.2% of 213 patients [14], Krobbuaban et al. recorded DVL in 12% of 550 subjects [15], Ezri et al. recorded DVL in 18% of 50 obese patients [16], Arunotai et al. documented DVL in 7.2% of 500 obese patients [17], and Komatsu et al. observed an incidence of DVL of 31% in 64 obese patients [8]. The literature primarily used the original Cormack and Lehane grading, with only grades 3 and 4 used to define DVL. When applying this criterion to our study, the incidence of DVL declined to 18 patients (20%). Ezri et al. [18] mentioned the use of external laryngeal maneuvers (ELM) in their study, whereas some studies did not clearly state whether ELM was used when recording the Cormack and Lehane grade, which can significantly alter the grade. Komatsu et al., in their study, avoided the use of ELM and reported a higher incidence of DVL (31%). Since the application of ELM cannot be standardized across all patients, we avoided its use in our study to maintain consistency in MCL grade recording. This may explain the variance in the incidence of DVL.

There was a higher incidence of DVL in the O group (21 patients, 46.7%) compared to the NO group (16 patients, 35.6%). However, the difference was not statistically significant (p > 0.05). Tingting et al., in a meta-analysis including 112,388 patients from nine studies, observed that obesity was associated with an increased risk of difficult intubation, difficult laryngoscopy, and a Mallampati score ≥ 3 in adult patients undergoing general surgical procedures [3]. However, a subgroup analysis showed no association between obesity and the risk of difficult intubation compared to non-obesity in cohort studies, which is consistent with our findings. Similarly, Ezri et al., in their study of 200 morbidly obese and 1,272 non-obese patients, found that obesity did not influence difficult laryngoscopy [18]. This suggests that there is no significant difference in the incidence of DVL between the obese and non-obese populations. We recommend that vigilant pre-operative airway assessments be applied to all patients, regardless of body weight, BMI, or obesity.

Modified Mallampati grading

In our study, grades 3 and 4 were considered predictors of DVL. We observed that 40 patients (44.4%) in our study population had an MMG score of 3 or 4. Only two patients in the entire population had an MMG grade 4; both were in the obese group and had DVL. In the NO group, only seven patients (39%) with DVL had an MMG score ≥ 3, with no significant association between MMG and MCL grade. In the O group, 13 patients (62%) with DVL showed a significant correlation with MMG (p < 0.05) (Table 6). MMG demonstrated high specificity in both groups: 85.1% in the O group and 70.8% in the NO group. The overall accuracy of MMG in both groups (O and NO) was 66.7% (Table 7). Vikas et al., in a study of 198 patients, found that MMG ≥ 3 had a specificity of 99.4% [19], while Ankalwar et al., in a study of 60 obese patients, recorded MMG specificity at 65.9% [20]. Ajinkya et al., in a study of 300 patients, found MMG ≥ 3 to have significant diagnostic validity [13]. Arunotai et al., in their study of 500 patients, also reported a high statistical significance of MMG ≥ 3 with DVL [17]. The MMG is a simple bedside clinical assessment test that is quick, easy to perform, and provides reliable results.

Neck circumference at the level of the thyroid

A large neck circumference indicates increased neck soft tissue mass and volume, which, under general anesthesia, can cause collapse and restrict the anterior displacement of soft tissue above the pharyngolaryngeal structures during direct laryngoscopy. This impedes direct laryngoscopy and results in inadequate exposure of the laryngeal inlet [16,17]. In the non-obese group, NCt was statistically non-significant in differentiating a DVL. In the obese group, the mean NCt of those with DVL was 38.75 ± 4.91 cm, but this was not statistically significant (p = 0.052). Our findings regarding NCt are consistent with those of Arunotai et al., who studied 500 patients and found that neck circumference had no statistical significance, concluding that NCt was a poor predictor of DVL [17]. Liaskou et al., in a study of 341 adult patients, examined neck circumference > 37.5 cm as a predictor of DVL and found that NCt had no statistical significance and was a poor single predictor of difficult laryngoscopy [9]. Similar findings were reported by Aylin et al., in a study of 120 patients, where neck circumference was not a statistically significant predictor for difficult laryngoscopy, even in the morbidly obese population [21]. Riad et al. had conflicting results, as neck circumference (NCt) was found to be statistically significant when considering a higher cut-off value of >42 cm, but it was associated with difficult intubation based on the intubation difficulty scale. The study population consisted of morbidly obese (BMI > 40 kg/m²) surgical patients [22].

Neck circumference at the level of the hyoid

Adhikari et al., in their study searching for an ultrasonographic airway index to predict a difficult airway, found a significant value of anterior neck soft tissue thickness above the hyoid bone [6]. This finding was further supported by the results observed by Yadav et al., where the skin-to-hyoid bone distance was a better predictor than other clinical tests [5]. Despite being comprehensive and requiring an operator learning curve, ultrasonographic airway assessment is not yet routinely practiced. However, a surrogate measurement for the ultrasound-derived soft tissue thickness above the hyoid bone can be devised by measuring the NCh bone. Neck circumference was measured around the mid-cervical spine and anterior neck at the level of the hyoid bone with the head in extension. In the non-obese group, the mean NCh of patients with DVL was 37.68 ± 3.02 cm, with no statistically significant association (p > 0.05). In the obese group, the mean NCh of patients with DVL was 43.32 ± 5.82 cm. The difference was not statistically significant (p = 0.054). Similar to NCt, NCh showed no significant difference in either group.

We observed that NCt and NCh had limited significance in differentiating a DVL from an EVL. The mean BMI of patients in the O group was 34 ± 3.9 kg/m². Patients enrolled in the study by Brodsky et al. had morbid obesity (BMI > 40 kg/m²), where NCt was found to be a good predictor of difficult laryngoscopic view with a cut-off value of 42 cm [23]. Neck circumference was found to have low sensitivity as a predictor of a difficult airway in a study by Liaskou et al. The accuracy of neck circumference only improved when a gender-specific cut-off value of 37.5 cm was applied for women [9]. It was observed that neck circumference as a predictor of DVL was confined to a population with morbid obesity, while the diagnostic utility in the general population is inconsistent and limited.

Neck circumference/thyromental distance ratio

The information on NCt and TMD as standalone indicators of DVL has been conflicting [17]. In 2011, Kim et al. utilized both indicators to formulate the neck circumference to TMD ratio (NCt/TMD). The NCt/TMD was devised based on the idea that obese patients with both larger neck circumference and shorter necks are more difficult to intubate than either of these factors alone [24]. In the NO group, patients with DVL had a mean NCt/TMD of 5.25 ± 1.33, which was statistically non-significant (p > 0.05). In the obese group, the mean NCt/TMD of patients with DVL was 5.3 ± 0.76, which was a statistically significant predictor (p < 0.05). ROC curve analysis in the obese group showed that a cut-off value of 5.135 had a sensitivity of 61.9% and a specificity of 58.3% (95% CI: 0.522 - 0.871). A study by Kim et al., which evaluated 123 obese and 125 non-obese patients, showed that NCt/TMD ≥ 5.0 had high sensitivity and specificity in predicting difficult intubation [24]. Similar findings were reported by Naim et al. in a study of 50 patients, where NCt/TMD ≥ 5.15 had a significant association with difficult intubation, showing high sensitivity and specificity (82%) [25]. Rose et al. published a comparison of NCt/TMD in predicting difficult intubation among 166 obese and 166 non-obese patients, where NCt/TMD ≥ 4.99 was a statistically significant variable in the obese group [26]. NCt/TMD can be a reliable bedside test for predicting a difficult airway, but only in the obese population.

Hyomental distance

The hyomental distance (HMD) is an estimate of the mandibular space that has been used to predict a DVL on its own. The HMD was measured in the neutral position (HMDn) of the head and at extension (HMDe), and the HMDR was the ratio of HMDe to HMDn. The findings were further evaluated, and the authors found similar results, with good predictive utility for DVL. In our study, the mean HMDR for those with DVL in the non-obese group was 1.1 ± 0.2. HMDR was statistically significant with a p-value <0.05. ROC curve analysis showed that an HMDR ≤ 1.23 had a sensitivity of 68.8% and specificity of 72.4% (95% CI: 0.607 - 0.936). In the obese population, the mean HMDR was 1.31 ± 0.1 for patients with EVL and 1.1 ± 0.2 for patients with DVL. The HMDR was statistically significant with a p-value < 0.05. ROC curve analysis in the obese group showed that an HMDR ≤ 1.20 had a sensitivity of 85.7% and specificity of 91.7% (95% CI: 0.808 - 1.000). Huh et al., in their evaluation of 213 adults undergoing general anesthesia, found similar results, with an HMDR threshold of 1.2 showing a sensitivity of 88% and specificity of 60% [14]. Ajinkya et al., in a study of 300 patients to evaluate HMDR as a predictor of difficult laryngoscopy, found results supportive of our findings, as an HMDR ≤ 1.2 was statistically significant [13]. The high specificity of HMDR in predicting a DVL was consistent with the findings of Rao et al., who studied 198 apparently normal patients and found that HMDR had 98% specificity in predicting a DVL [27]. Our observation is consistent with the finding that an HMDR ≤ 1.2 has a high specificity of 98.9%, as reported by Vikas et al. in their evaluation of 198 patients [19]. HMDR has shown consistent performance in predicting a DVL, irrespective of BMI or obesity. Thus, HMDR can be a reliable bedside airway assessment tool.

A good predictive index should possess both high sensitivity and specificity. However, when it comes to potentially maximizing patient safety and avoiding complications associated with laryngoscopy and failed intubation, minimizing false negative predictions is preferable to minimizing false positives. Although MMG was statistically significant only in the obese group, an MMG ≥ 3 demonstrated high specificity in both groups, with an accuracy of 66.7% in each group. The NCt/TMD, with a cut-off value of 5.135, showed good diagnostic value in the obese population. An HMDR ≤ 1.20 in the obese group and ≤1.23 in the non-obese group was statistically significant, with high sensitivity and specificity. HMDR was the only index that was a statistically significant predictor of a DVL in both groups, with higher specificity than all other indices.

Limitations

We encountered several limitations in our study. The ASA status and comorbid conditions of the patient population were not considered. We defined MCL grades 2b, 3, or 4 as indicators of DVL without any external laryngeal manipulation (ELM). The application of ELM in many clinical scenarios can potentially improve the MCL grade and reduce the difficulty of endotracheal intubation. A small sample size is also a limitation of the study.

## Conclusions

The incidence of DVL in the obese population is higher compared to the non-obese population, but the difference is not statistically significant. We suggest that a vigilant preoperative airway assessment should be applied to all patients, regardless of body weight, BMI, or obesity. Based on the results of our study, we conclude that HMDR is a better predictor of DVL in both obese and non-obese populations, independent of BMI. However, MMG and the NCt/TMD ratio were predictors of DVL only in the obese population. NCt and NCh have minimal utility as single predictors of DVL.

## References

[1] Matioc AA (2018). An anesthesiologist’s perspective on the history of basic airway management: the “progressive” era, 1904 to 1960. Anesthesiology.

[2] Detsky ME, Jivraj N, Adhikari NK (2019). Will this patient be difficult to intubate?: the rational clinical examination systematic review. J Am Med Assoc.

[3] Wang T, Sun S, Huang S (2018). The association of body mass index with difficult tracheal intubation management by direct laryngoscopy: a meta-analysis. BMC Anesthesiol.

[4] Heidegger T (2021). Management of the difficult airway. N Engl J Med.

[5] Yadav NK, Rudingwa P, Mishra SK, Pannerselvam S (2019). Ultrasound measurement of anterior neck soft tissue and tongue thickness to predict difficult laryngoscopy - an observational analytical study. Indian J Anaesth.

[6] Adhikari S, Zeger W, Schmier C (2011). Pilot study to determine the utility of point-of-care ultrasound in the assessment of difficult laryngoscopy. Acad Emerg Med.

[7] Kamranmanesh MR, Jafari AR, Gharaei B, Aghamohammadi H, Poor Zamany NKM, Kashi AH (2013). Comparison of acromioaxillosuprasternal notch index (a new test) with modified Mallampati test in predicting difficult visualization of larynx. Acta Anaesthesiol Taiwan.

[8] Komatsu R, Sengupta P, Wadhwa A, Akça O, Sessler DI, Ezri T, Lenhardt R (2007). Ultrasound quantification of anterior soft tissue thickness fails to predict difficult laryngoscopy in obese patients. Anaesth Intensive Care.

[9] Liaskou C, Vouzounerakis E, Moirasgenti M, Trikoupi A, Staikou C (2014). Anatomic features of the neck as predictive markers of difficult direct laryngoscopy in men and women: a prospective study. Indian J Anaesth.

[10] Rao ST, Gowda V, Reddy RV (2013). Hyomental distance ratio as a diagnostic predictor of difficult laryngoscopy. Indian J Appl Res.

[11] Samsoon GL, Young JR (1987). Difficult tracheal intubation: a retrospective study. Anaesthesia.

[12] Yentis SM, Lee DJ (1998). Evaluation of an improved scoring system for the grading of direct laryngoscopy. Anaesthesia.

[13] Bhosle A, Bhosle P, Aphale S (2015). Hyomental distance ratio & prediction of difficult laryngoscopy with Cormack Lehane grading. J Res Med Den Sci.

[14] Huh J, Shin HY, Kim SH, Yoon TK, Kim DK (2009). Diagnostic predictor of difficult laryngoscopy: the hyomental distance ratio. Anesth Analg.

[15] Krobbuaban B, Diregpoke S, Kumkeaw S, Tanomsat M (2005). The predictive value of the height ratio and thyromental distance: four predictive tests for difficult laryngoscopy. Anesth Analg.

[16] Ezri T, Gewürtz G, Sessler DI, Medalion B, Szmuk P, Hagberg C, Susmallian S (2003). Prediction of difficult laryngoscopy in obese patients by ultrasound quantification of anterior neck soft tissue. Anaesthesia.

[17] Siriussawakul A, Rattana-arpa S, Jirachaipitak S, Chatsiriphattana A, Nimmannit A, Wong-in N (20161). The performance of the neck circumference for a difficult laryngoscopy in obese patients. J Med Assoc Thai.

[18] Ezri T, Medalion B, Weisenberg M, Szmuk P, Warters RD, Charuzi I (2003). Increased body mass index per se is not a predictor of difficult laryngoscopy. Can J Anaesth.

[19] Vikas KN, Vinay R (2014). Comparison of Hyomental Distance Ratio with the modified Mallampati test for accurately predicting difficult visualization of the larynx. Int J Res Health Sci.

[20] Ankalwar VR, Patel M, Tirpude NG (2019). Neck circumference to thyromental distance ratio: Is a reliable predictor of difficult intubation in obese patients?. Indian J Clin Anaesth.

[21] Özdilek A, Beyoglu CA, Erbabacan ŞE, Ekici B, Altındaş F, Vehid S, Köksal GM (2018). Correlation of neck circumference with difficult mask ventilation and difficult laryngoscopy in morbidly obese patients: an observational study. Obes Surg.

[22] Riad W, Vaez MN, Raveendran R, Tam AD, Quereshy FA, Chung F, Wong DT (2016). Neck circumference as a predictor of difficult intubation and difficult mask ventilation in morbidly obese patients: a prospective observational study. Eur J Anaesthesiol.

[23] Brodsky JB, Lemmens HJ, Brock-Utne JG, Vierra M, Saidman LJ (2002). Morbid obesity and tracheal intubation. Anesth Analg.

[24] Kim WH, Ahn HJ, Lee CJ, Shin BS, Ko JS, Choi SJ, Ryu SA (2011). Neck circumference to thyromental distance ratio: a new predictor of difficult intubation in obese patients. Br J Anaesth.

[25] Abdel Naim HE, Mohamed SA, Soaida SM, Eltrabily HH (2014). The importance of neck circumference to thyromental distance ratio (NC/TM) as a predictor of difficult intubation in obstructive sleep apnea (OSA) patients. Egypt J Anaesth.

[26] Rose N (2017). Comparison of difficult intubation and neck circumference to thyromental distance ratio, in obese and non-obese: a clinical study. J Med Sci Clin Res.

[27] Safavi M (2016). Prediction of difficult laryngoscopy in pregnant women undergoing cesarean section using the Hyomental distance in fully extended and neutral position of neck, in comparison with four usual bedside tests: a prospective blinded study. J Anaesth and Surg.

